# Supplementary figures and images for: Cryo-EM ligand building using AlphaFold3-like model and molecular dynamics
Source: PLoS Comput Biol. 2025 Aug 11;21(8):e1013367. doi: 10.1371/journal.pcbi.1013367 (PMC12364316; doi:10.1371/journal.pcbi.1013367)

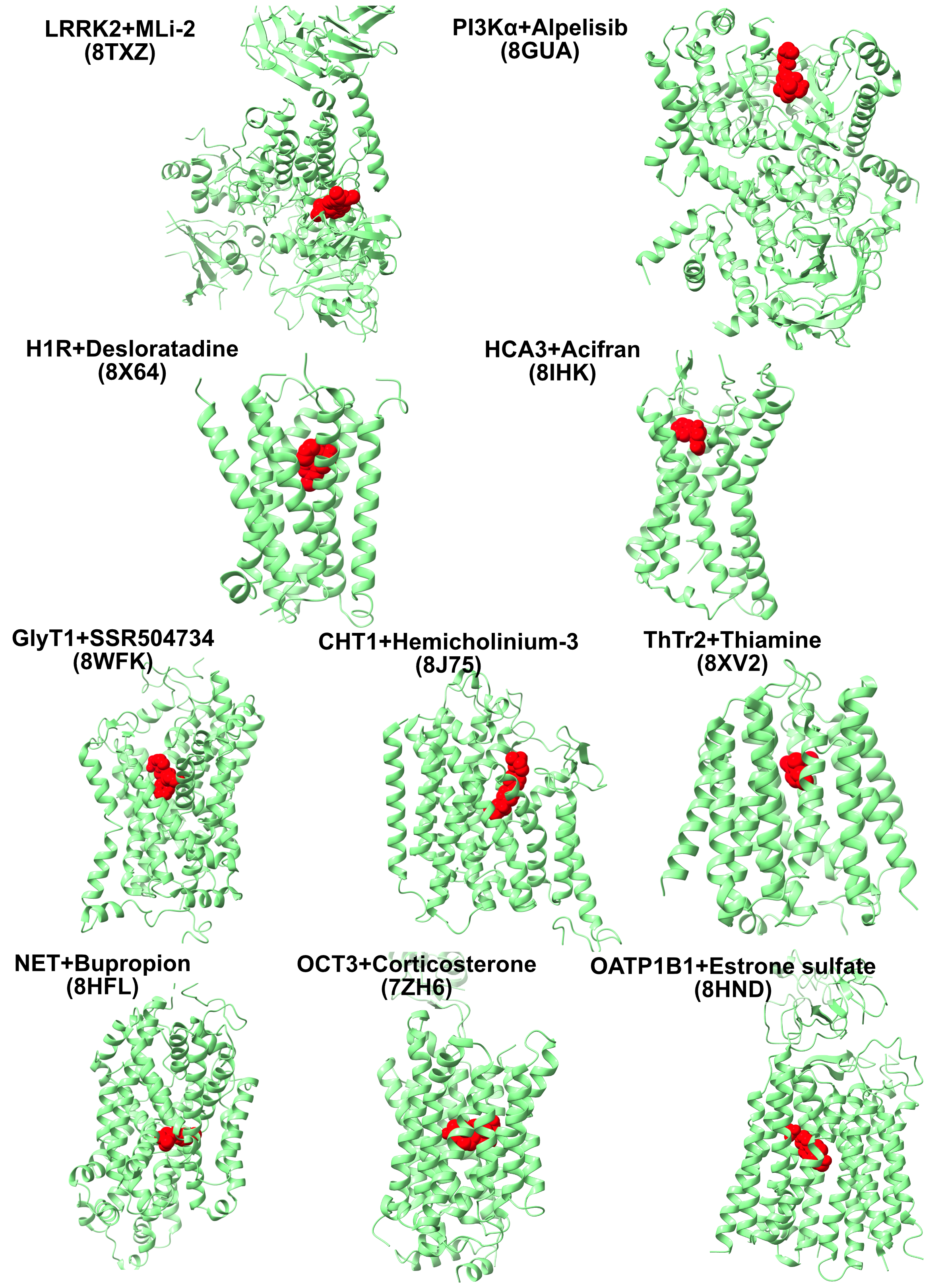

Supplement: S1 Fig — PDB IDs are described in brackets after the names of each protein. Red sphere shows the ligands bound to the proteins. (TIFF) [file pcbi.1013367.s002.tiff]

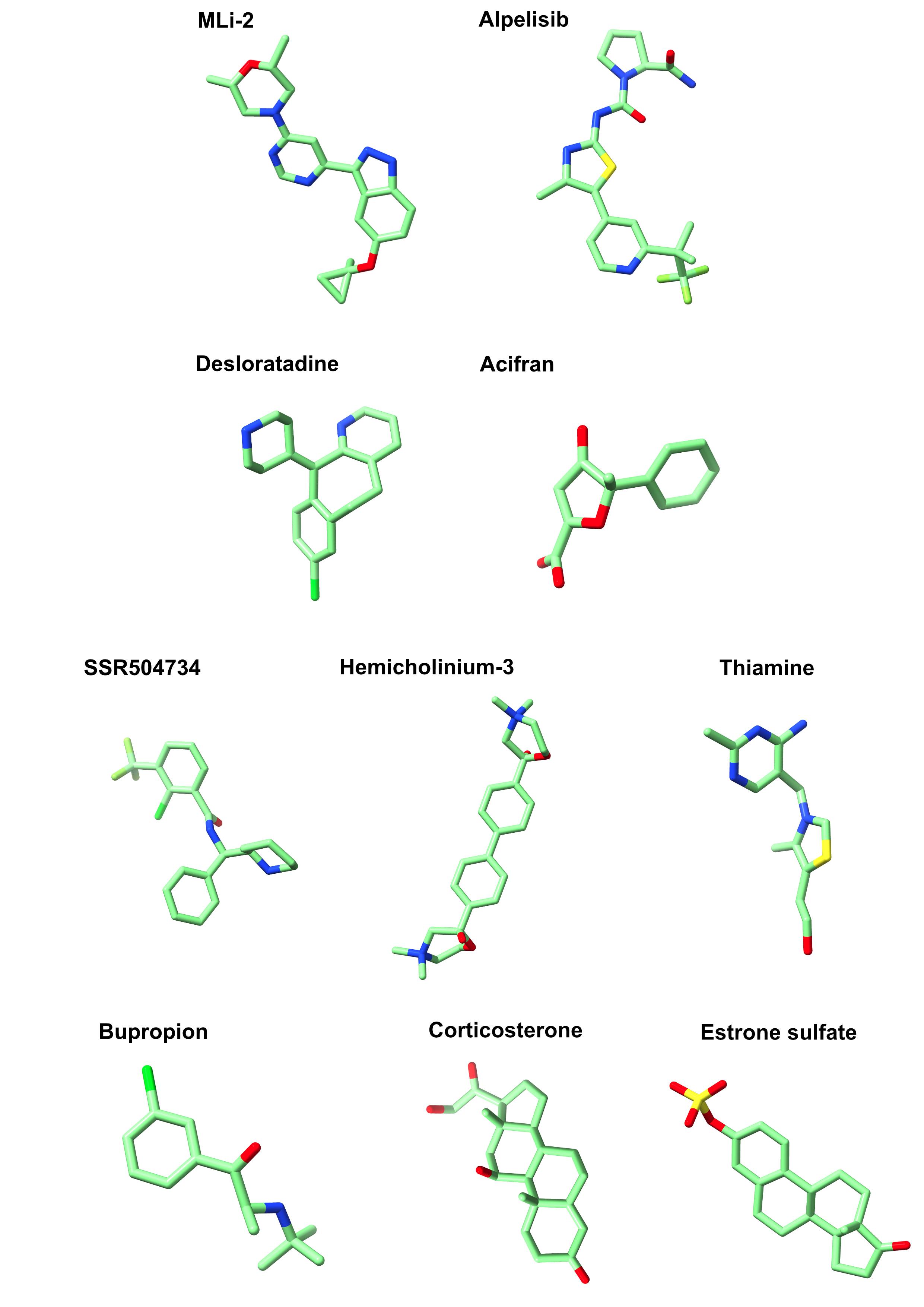

Supplement: S2 Fig — (TIFF) [file pcbi.1013367.s003.tiff]

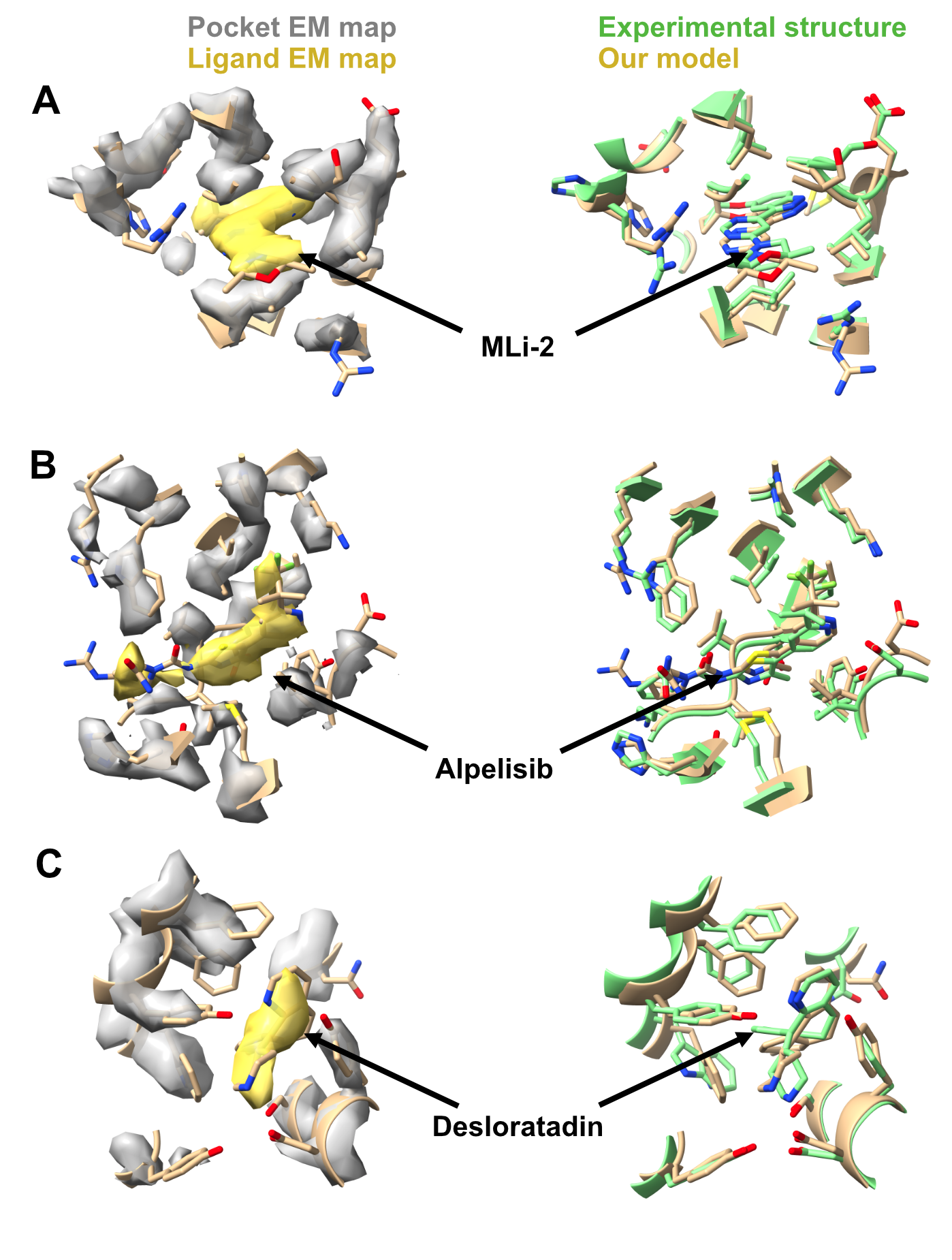

Supplement: S3 Fig — (Left) Initial Chai-1 prediction of the binding pocket for the, leucine-rich repeat kinase 2+MLi-2 (A), phosphoinositide 3-kinase α+alpelisib (B), and histamine H1 receptor+desloratadine (C) systems. Cryo-EM densities for the ligand (yellow) and pocket protein residues (silver) are shown in transparent. (Middle) Predicted structure (yellow) along with the experimental structure (green). Alignments were done using the entire protein in ChimeraX Matchmaker module. Chai-1 prediction of all the 5 possible binding sites can be found in S4, S5 and S6 Figs. (TIFF) [file pcbi.1013367.s004.tiff]

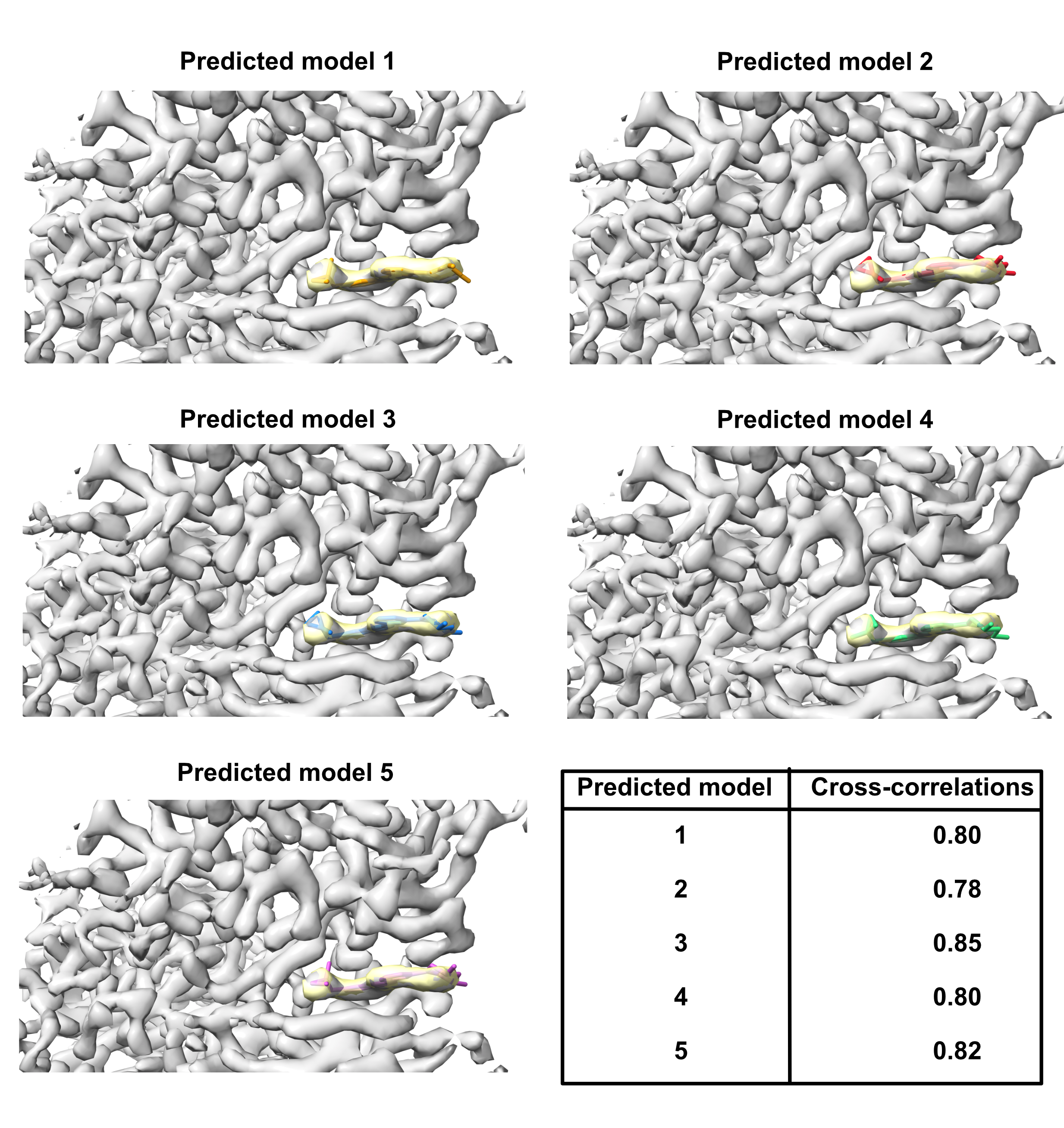

Supplement: S4 Fig — Cryo-EM densities for the ligand (yellow) and pocket protein residues (silver) are shown in transparent. Cross-correlation values for the ligands are shown in the table below. (TIFF) [file pcbi.1013367.s005.tiff]

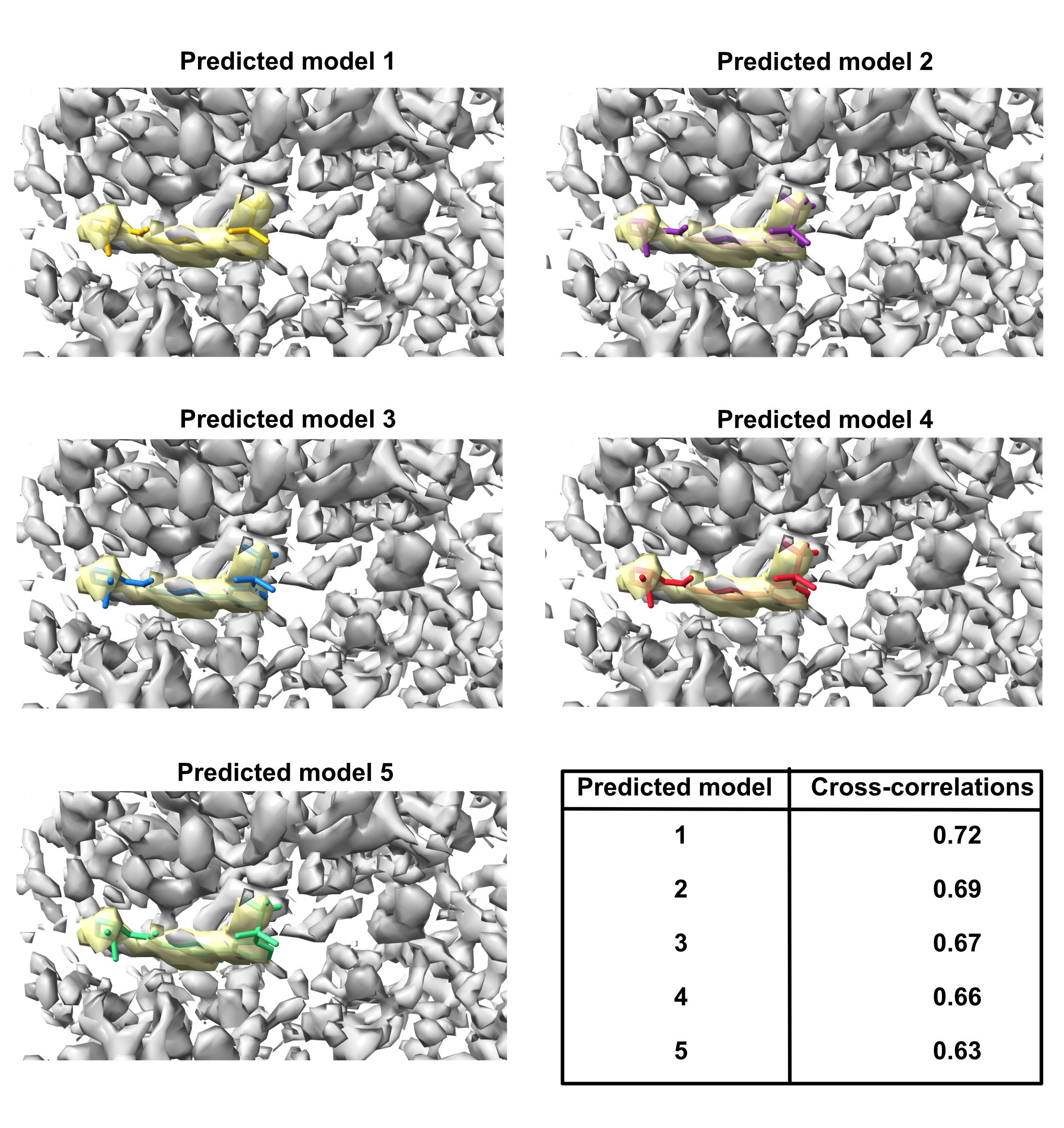

Supplement: S5 Fig — (TIFF) [file pcbi.1013367.s006.tiff]

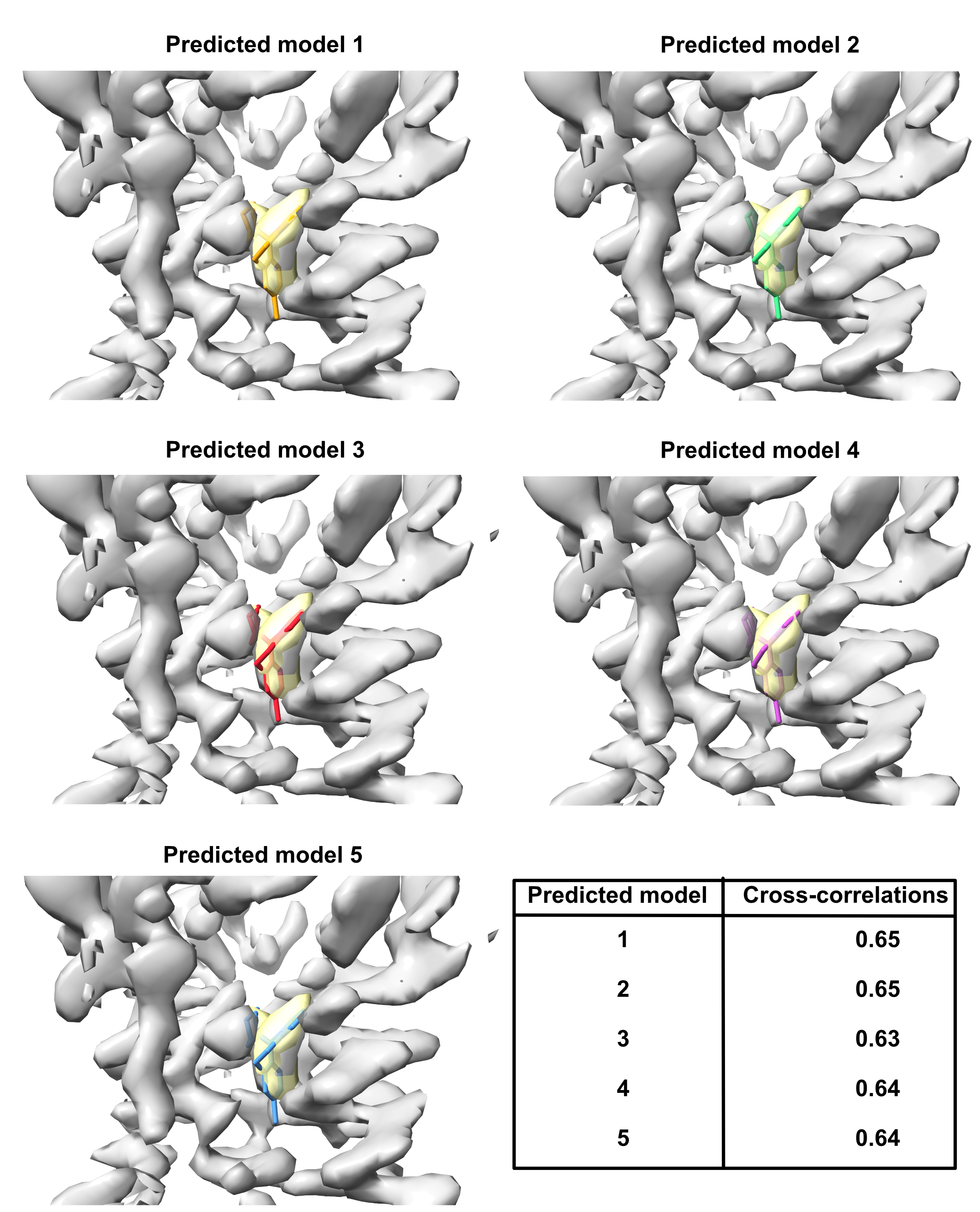

Supplement: S6 Fig — Cryo-EM densities for the ligand (yellow) and pocket protein residues (silver) are shown in transparent. Cross-correlation values for the ligands are shown in the table below. (TIFF) [file pcbi.1013367.s007.tiff]

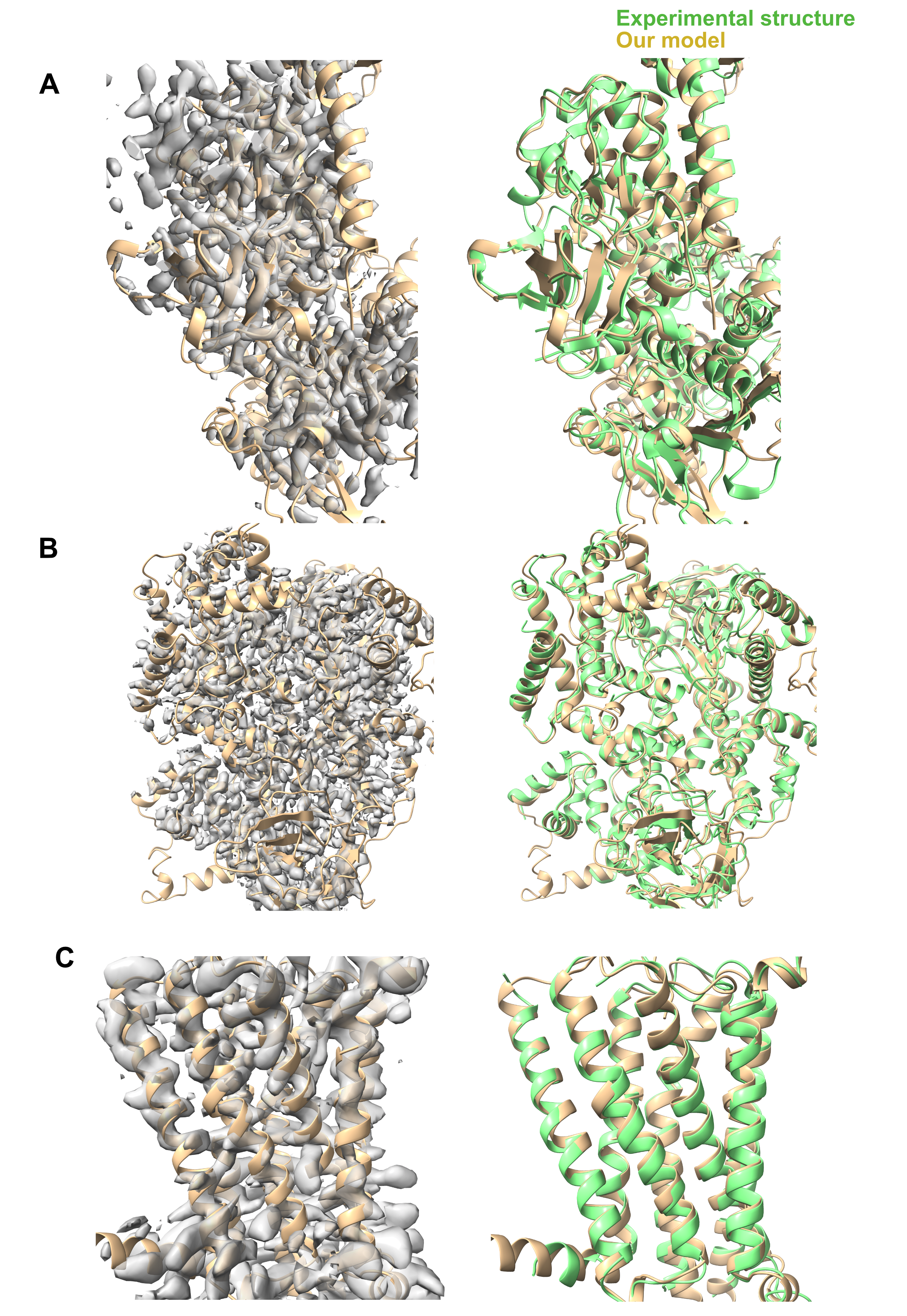

Supplement: S7 Fig — Predicted structure (yellow) along with the experimental structure (green) are shown on the right. (TIFF) [file pcbi.1013367.s008.tiff]

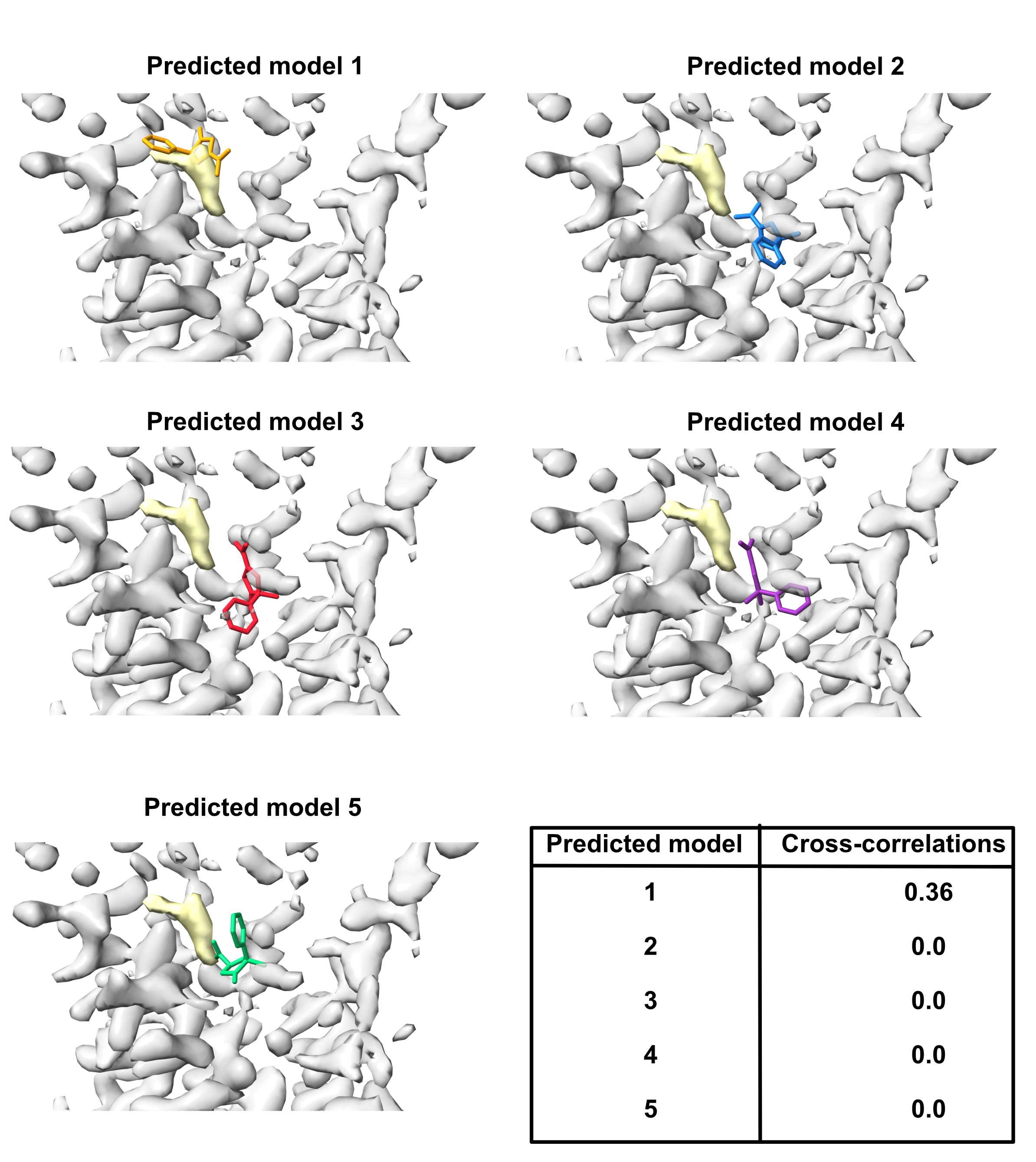

Supplement: S8 Fig — Cryo-EM densities for the ligand (yellow) and pocket protein residues (silver) are shown in transparent. Cross-correlation values for the ligands are shown in the table below. (TIFF) [file pcbi.1013367.s009.tiff]

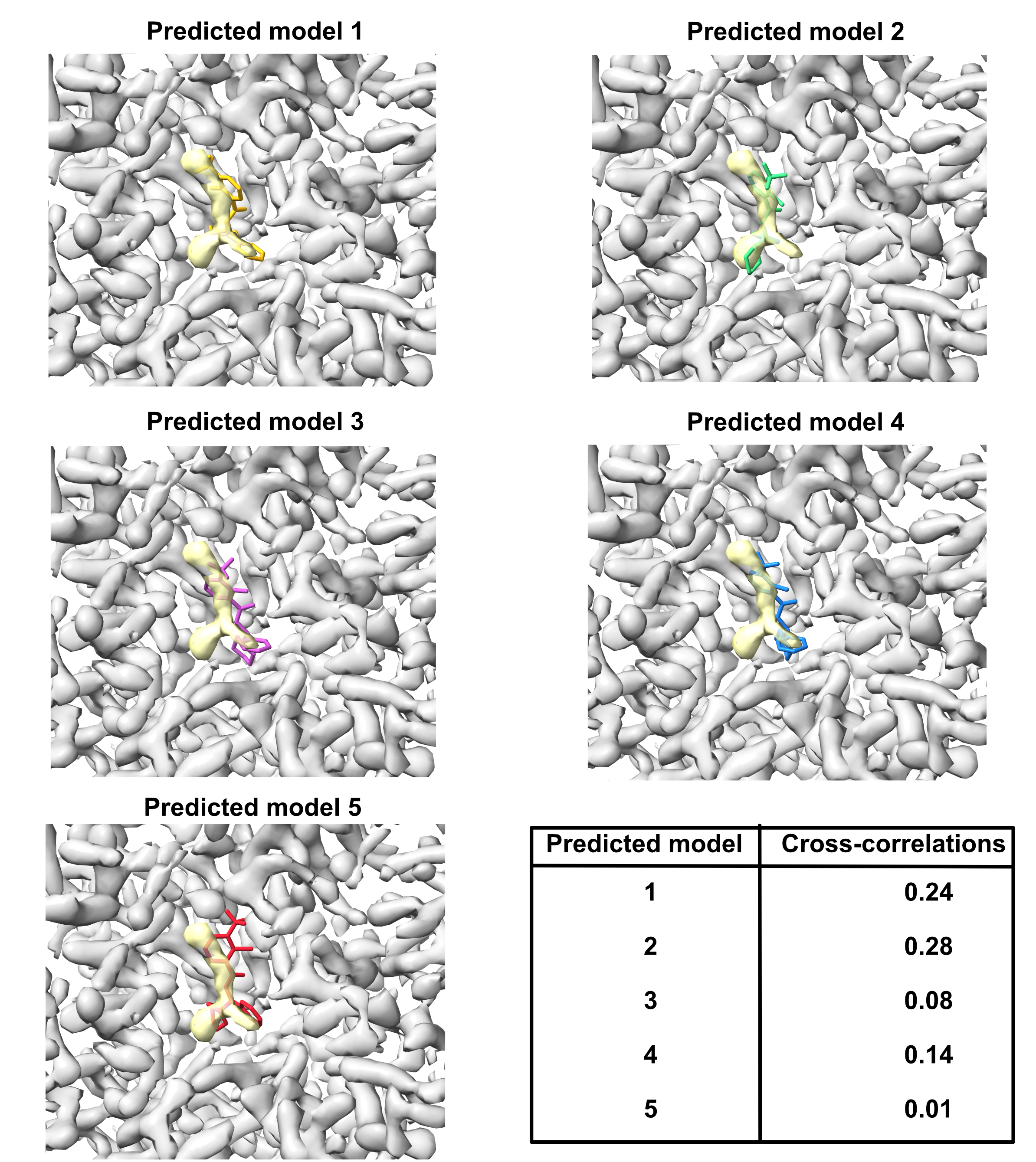

Supplement: S9 Fig — Cryo-EM densities for the ligand (yellow) and pocket protein residues (silver) are shown in transparent. Cross-correlation values for the ligands are shown in the table below. (TIFF) [file pcbi.1013367.s010.tiff]

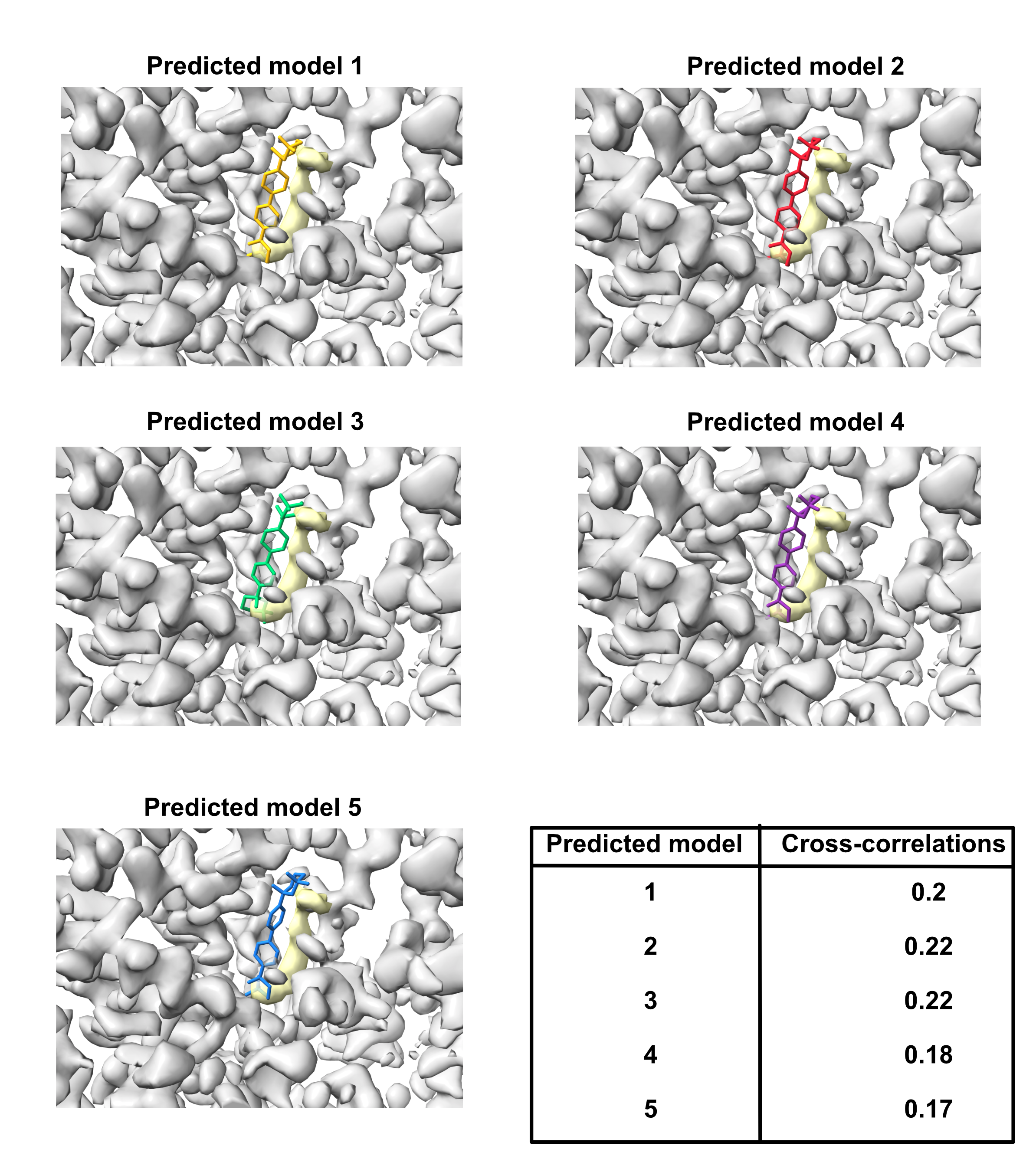

Supplement: S10 Fig — Cryo-EM densities for the ligand (yellow) and pocket protein residues (silver) are shown in transparent. Cross-correlation values for the ligands are shown in the table below. (TIFF) [file pcbi.1013367.s011.tiff]

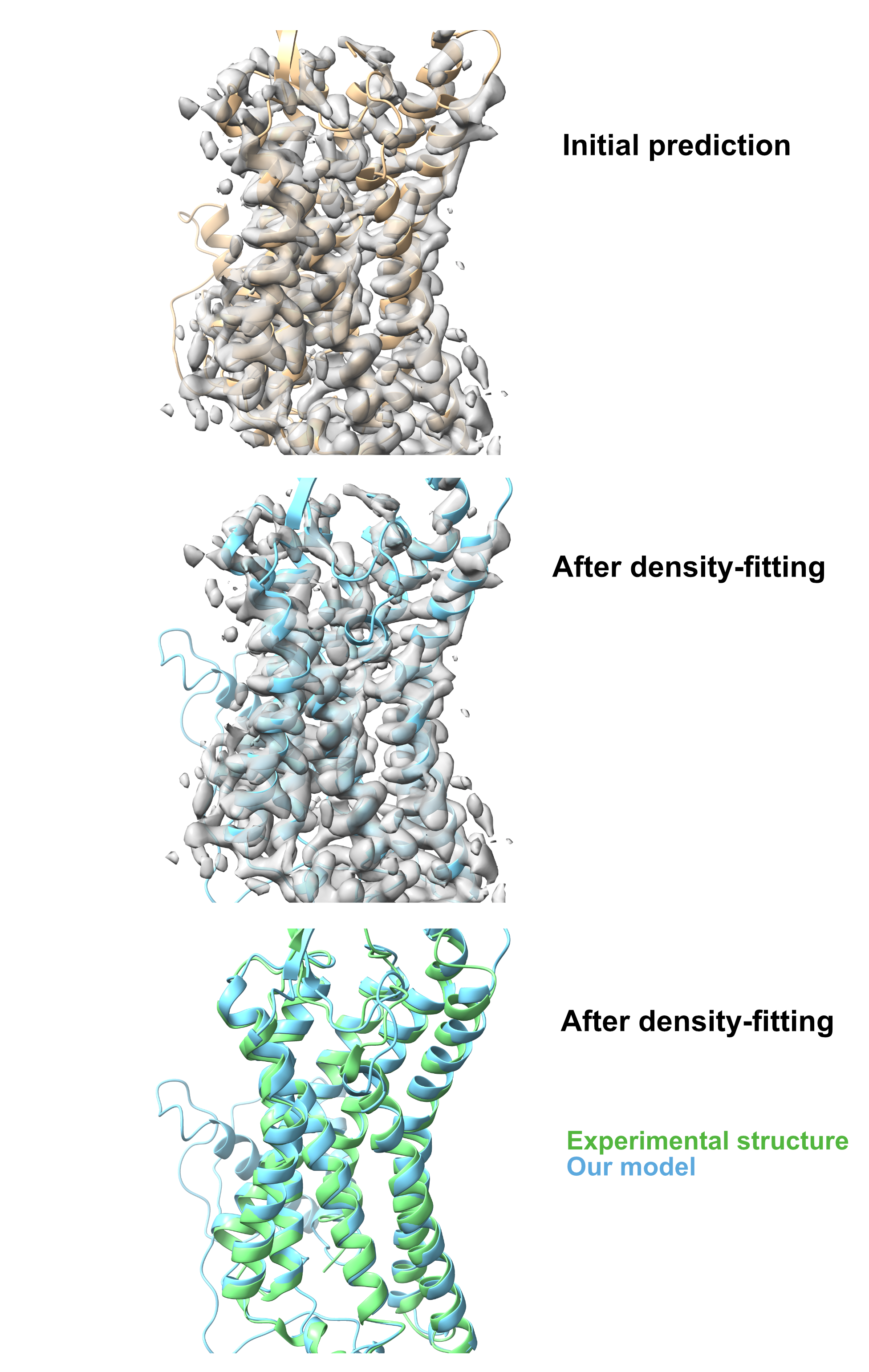

Supplement: S11 Fig — (TIFF) [file pcbi.1013367.s012.tiff]

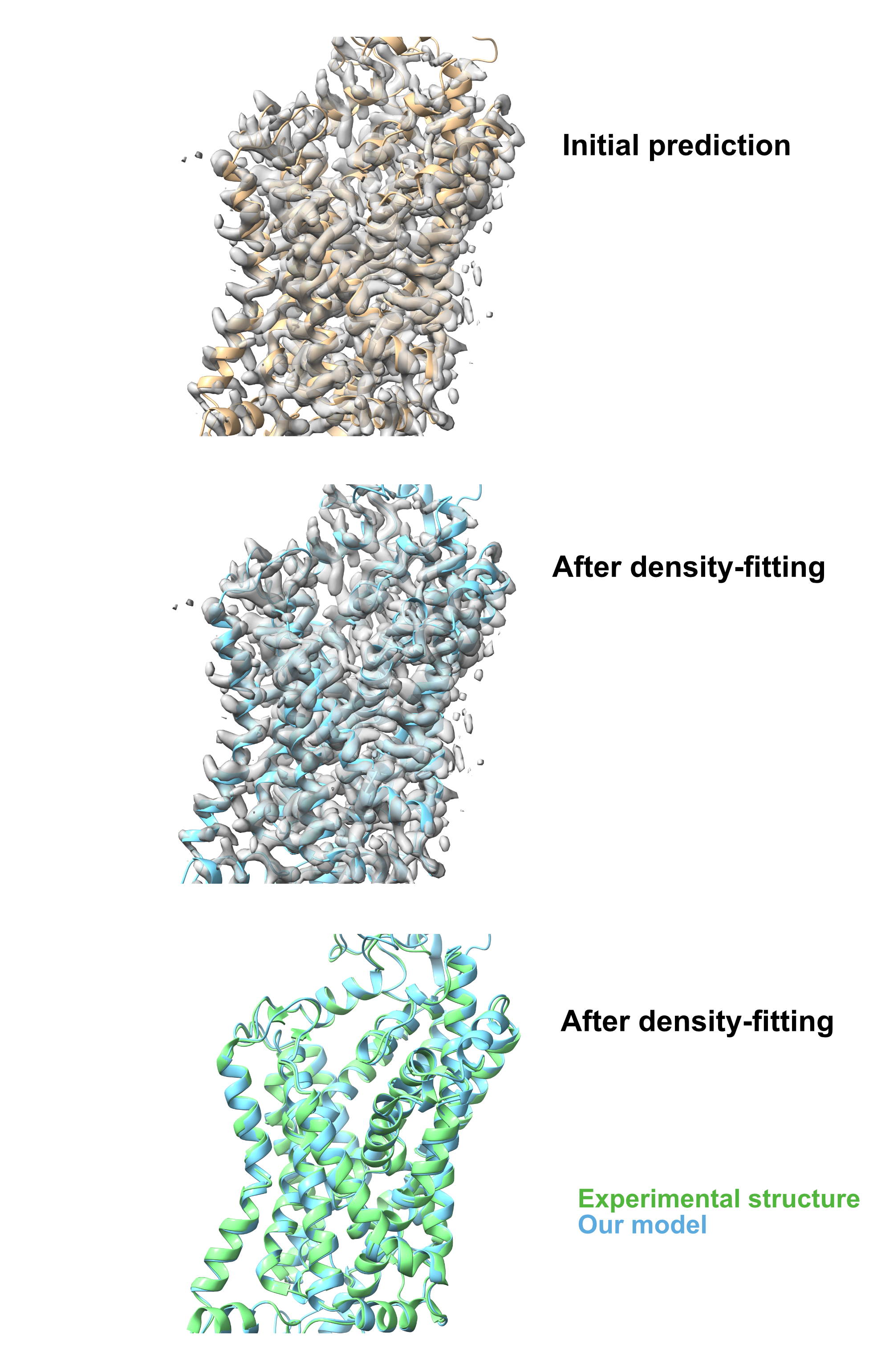

Supplement: S12 Fig — (TIFF) [file pcbi.1013367.s013.tiff]

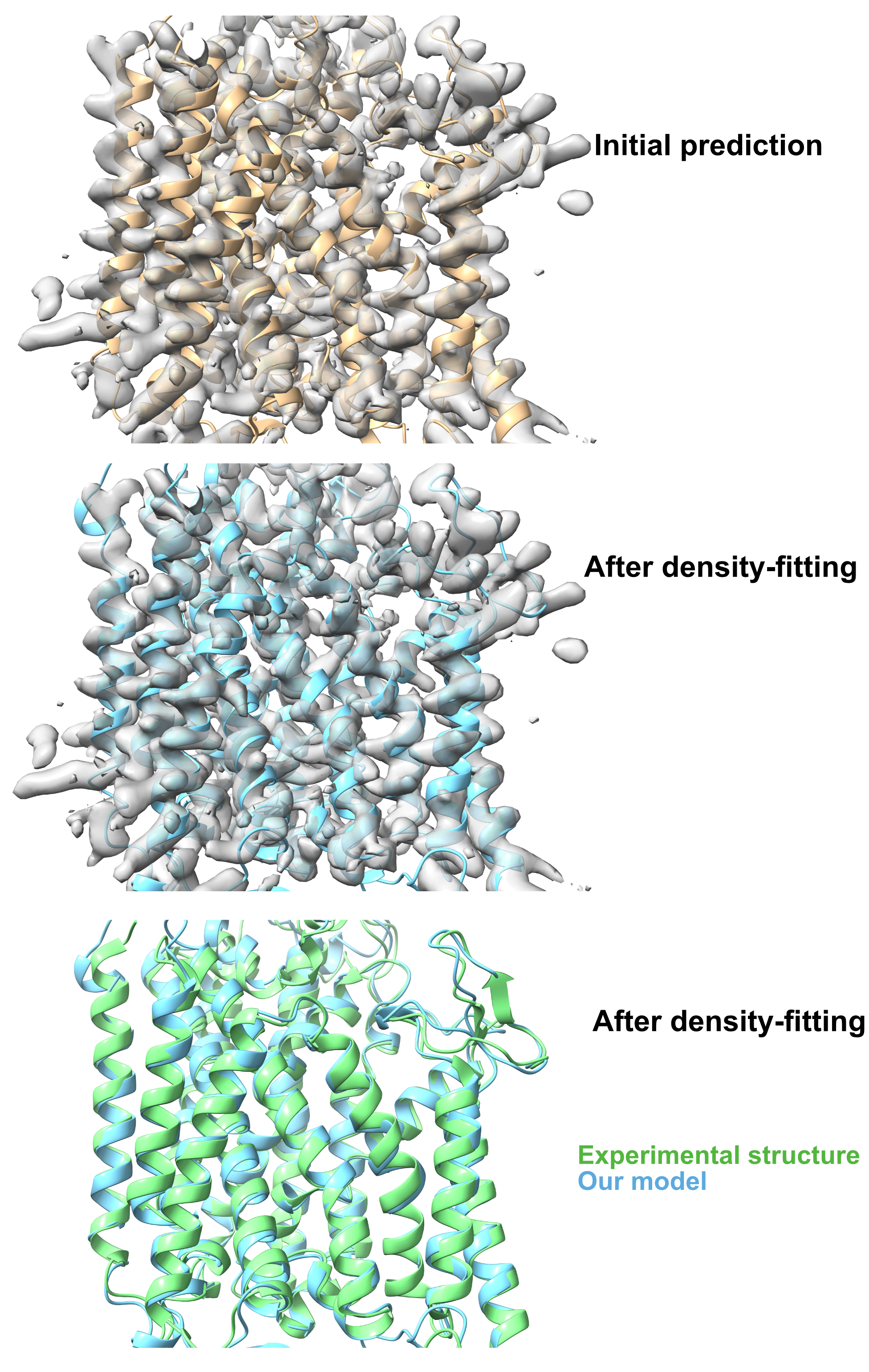

Supplement: S13 Fig — (TIFF) [file pcbi.1013367.s014.tiff]

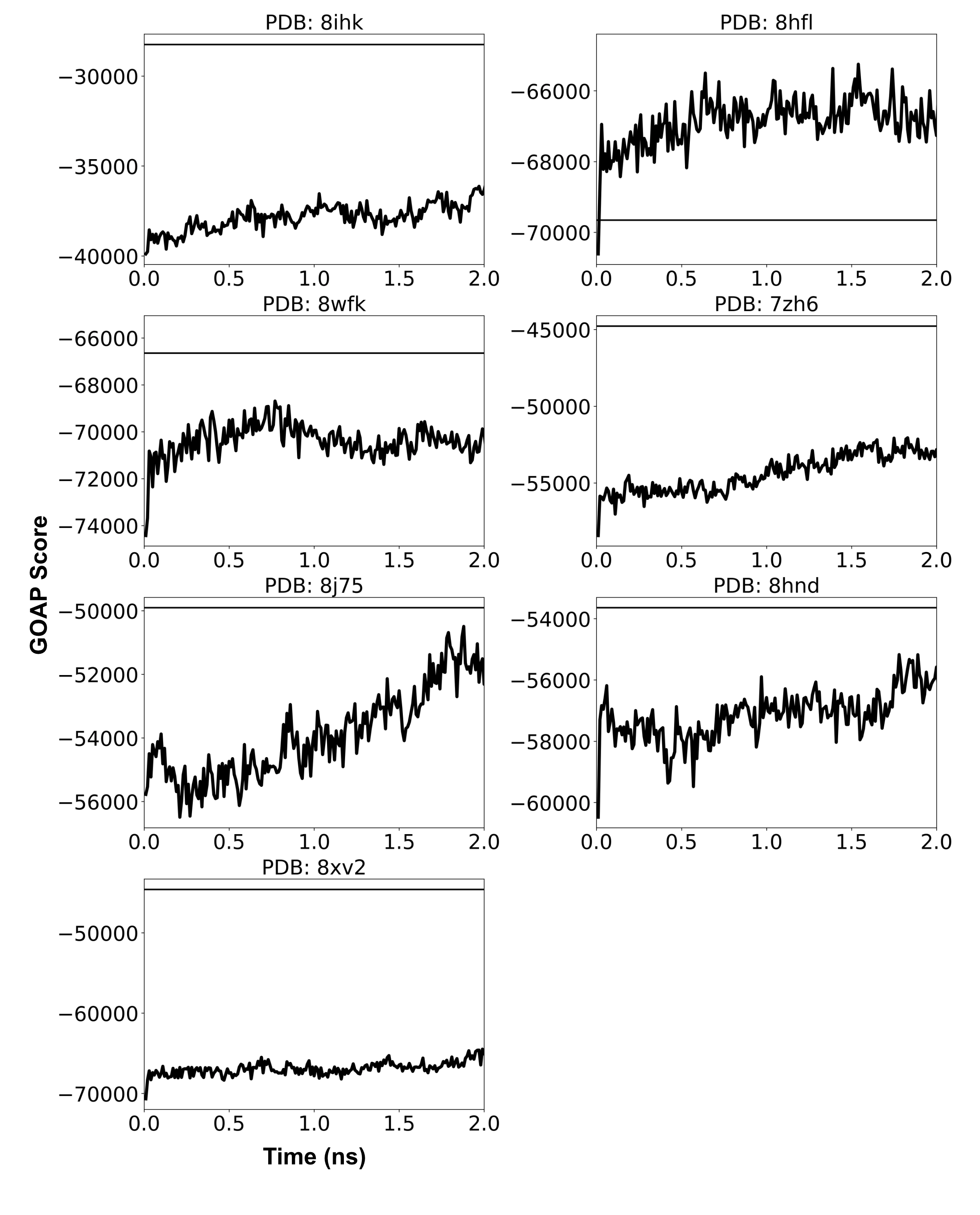

Supplement: S14 Fig — GOAP score for the experimental structure is depicted in the horizontal line. Lower the GOAP score, better the structure quality of the protein. (TIFF) [file pcbi.1013367.s015.tiff]

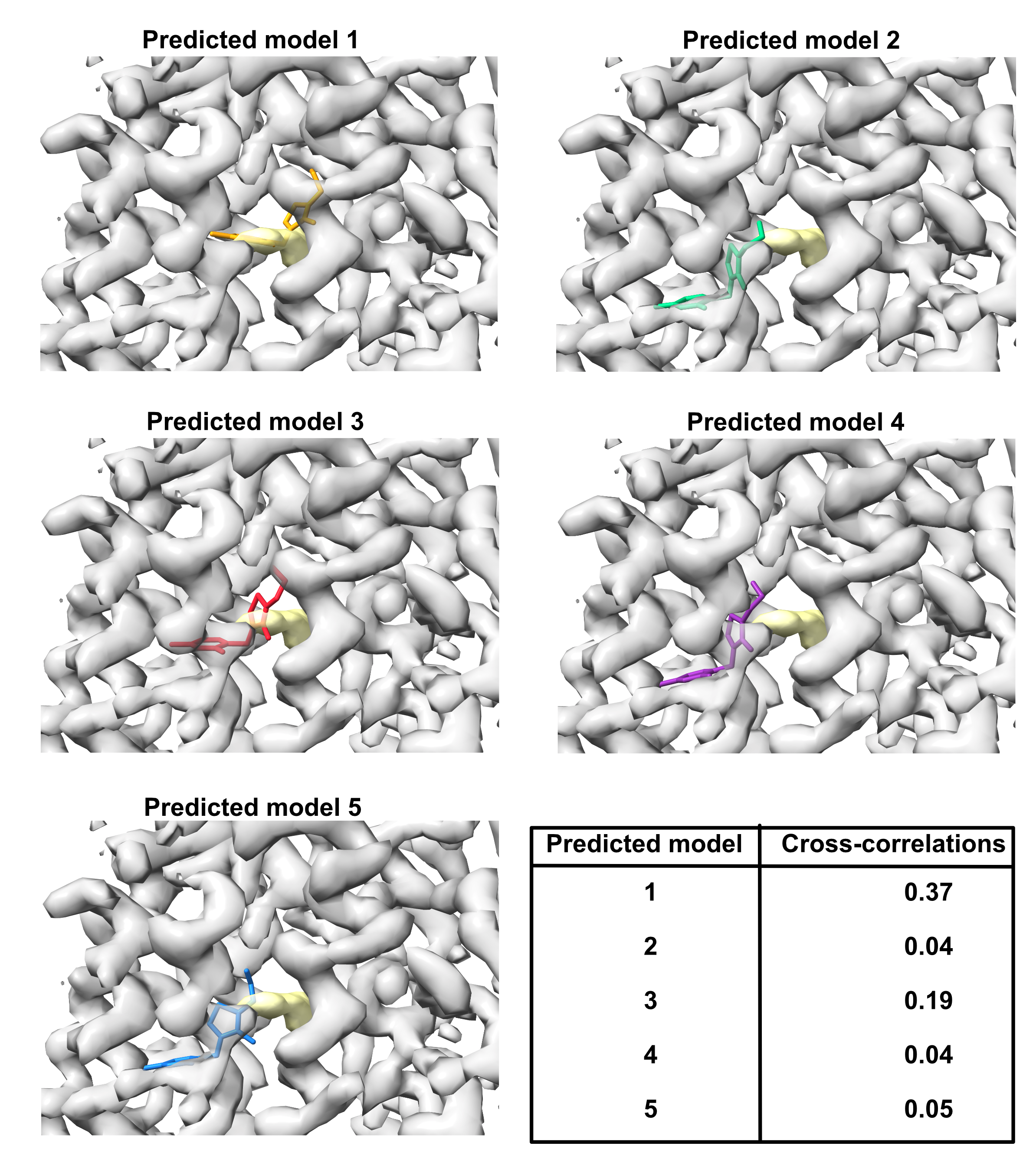

Supplement: S15 Fig — Cryo-EM densities for the ligand (yellow) and pocket protein residues (silver) are shown in transparent. Cross-correlation values for the ligands are shown in the table below. (TIFF) [file pcbi.1013367.s016.tiff]

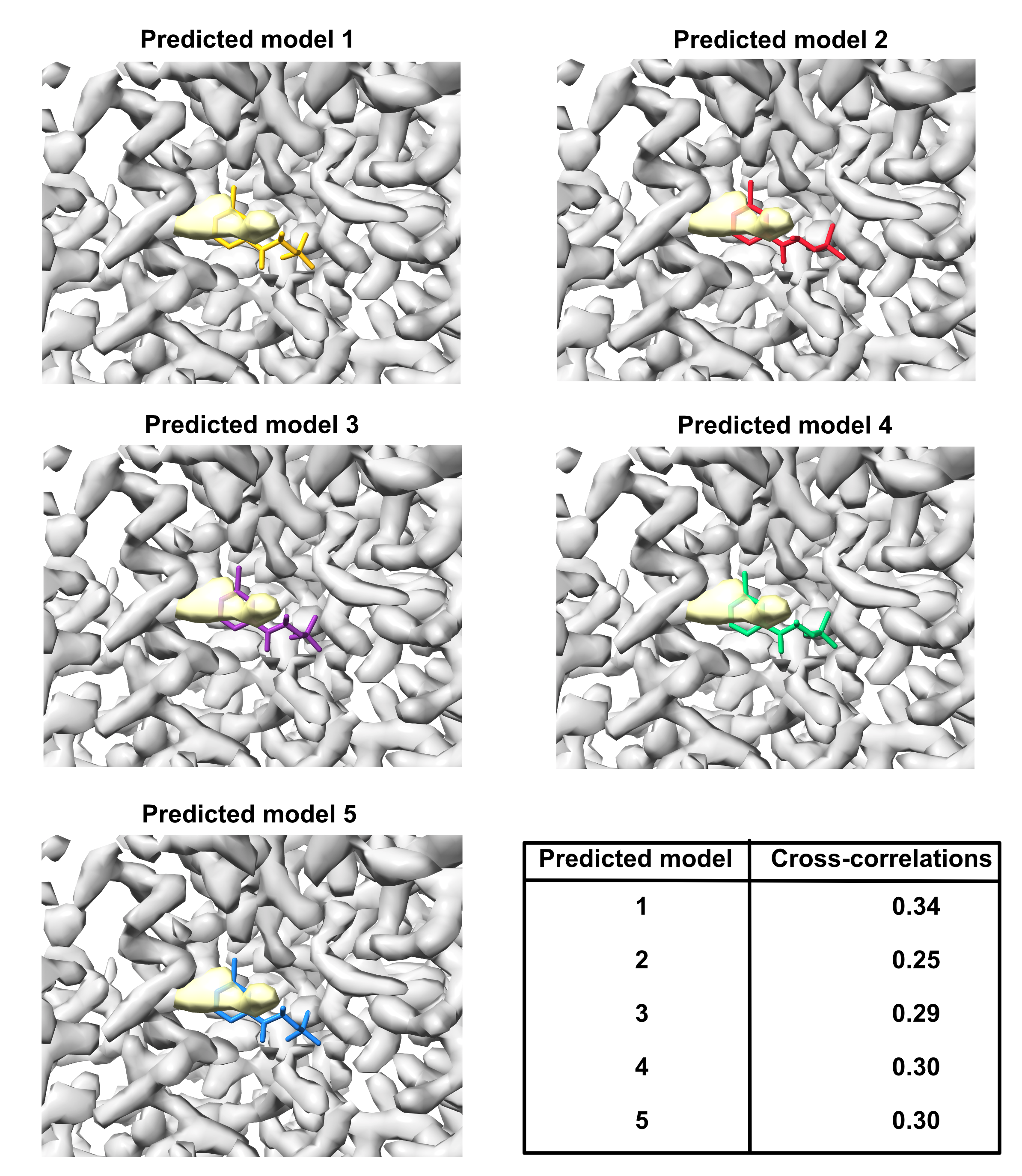

Supplement: S16 Fig — Cryo-EM densities for the ligand (yellow) and pocket protein residues (silver) are shown in transparent. Cross-correlation values for the ligands are shown in the table below. (TIFF) [file pcbi.1013367.s017.tiff]

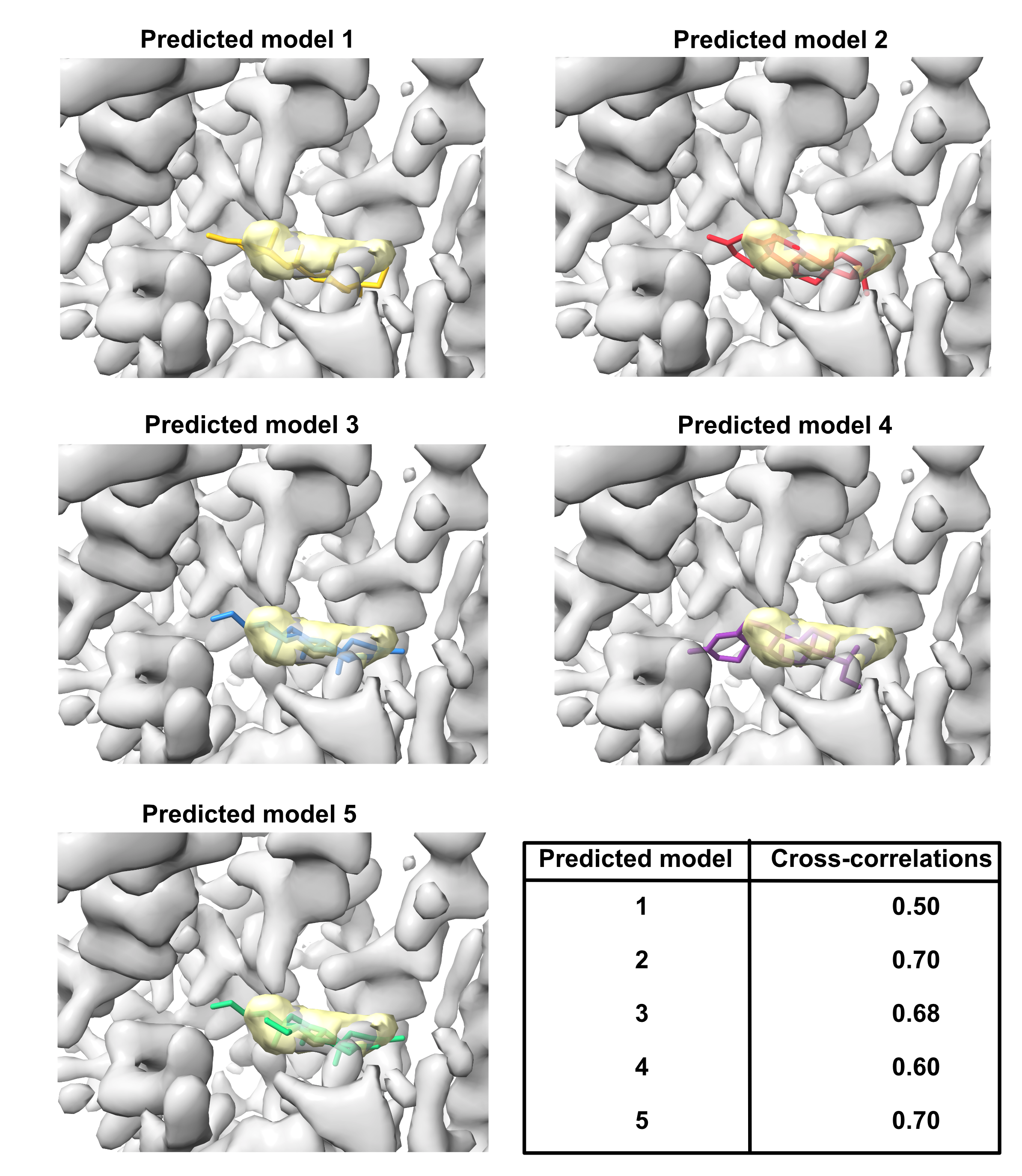

Supplement: S17 Fig — Cryo-EM densities for the ligand (yellow) and pocket protein residues (silver) are shown in transparent. Cross-correlation values for the ligands are shown in the table below. (TIFF) [file pcbi.1013367.s018.tiff]
